# Supplementary material for: Heterologous expression, purification, and biochemical characterization of protease 3075 from Cohnella sp. A01
Source: PLoS One. 2024 Dec 16;19(12):e0310910. doi: 10.1371/journal.pone.0310910 (PMC11649109; doi:10.1371/journal.pone.0310910)
Supplement: S1 Fig — M: molecular size marker, 1: genomic DNA extracted from Cohnella sp. A01, 2: circular plasmid, 3: Digested recombinant plasmid, 4: PCR product of the desired gene, 5: enzymatic digestion of recombinant plasmid, 6: empty digested vector with Nde I enzyme, 7: PCR colony, 8: PCR plasmid. (PPTX) [file pone.0310910.s001.pptx]

## Slide 1
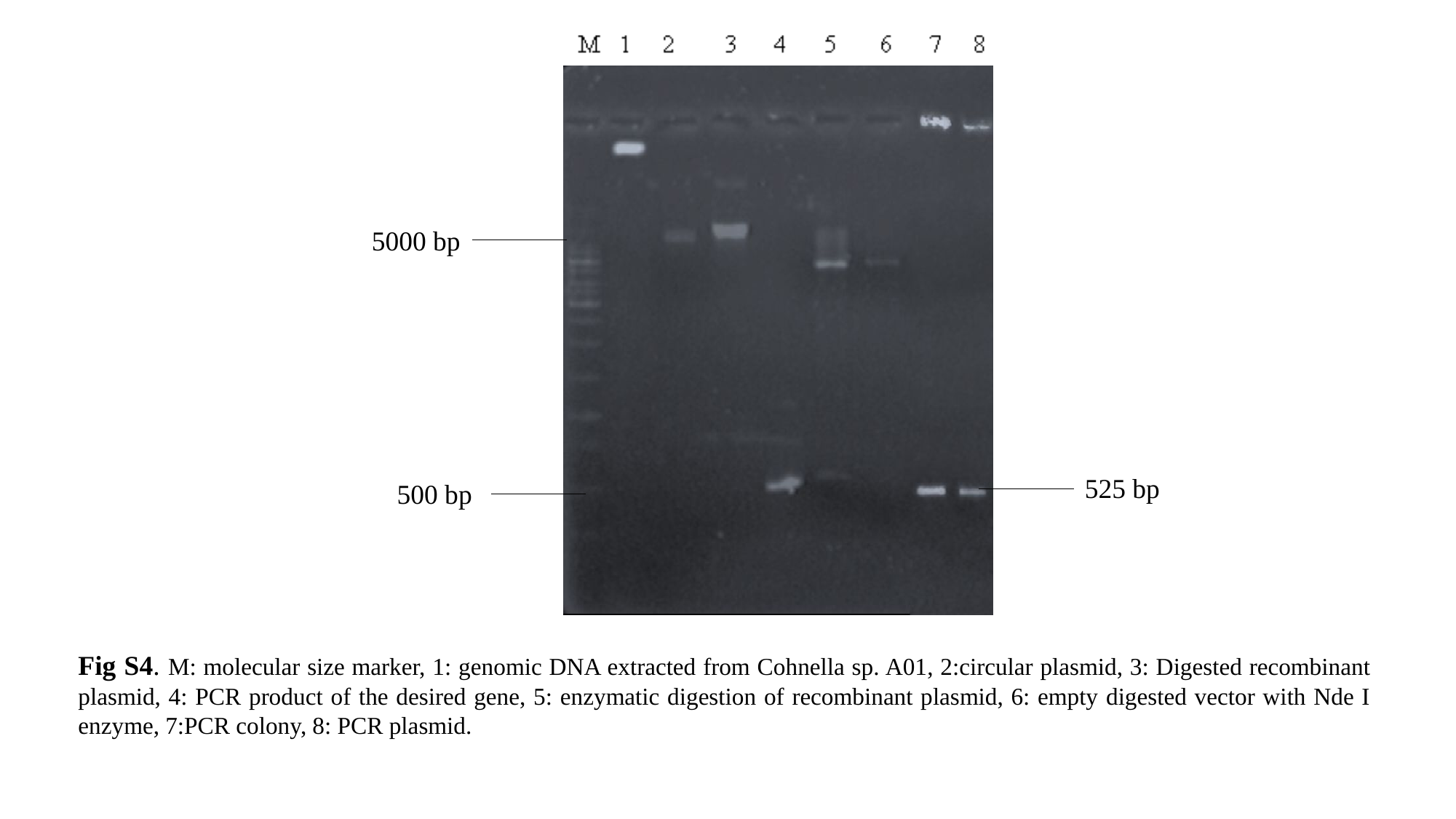

5000 bp
525 bp
500 bp
Fig S4. M: molecular size marker, 1: genomic DNA extracted from Cohnella sp. A01, 2:circular plasmid, 3: Digested recombinant plasmid, 4: PCR product of the desired gene, 5: enzymatic digestion of recombinant plasmid, 6: empty digested vector with Nde I enzyme, 7:PCR colony, 8: PCR plasmid.
