# Supplementary material for: Heterologous expression, purification, and biochemical characterization of protease 3075 from Cohnella sp. A01
Source: PLoS One. 2024 Dec 16;19(12):e0310910. doi: 10.1371/journal.pone.0310910 (PMC11649109; doi:10.1371/journal.pone.0310910)
Supplement: S2 Fig — Yellow arrows represent beta sheets, and pink cylinders represent alpha helix, which are connected by coils. (PPTX) [file pone.0310910.s002.pptx]

## Slide 1
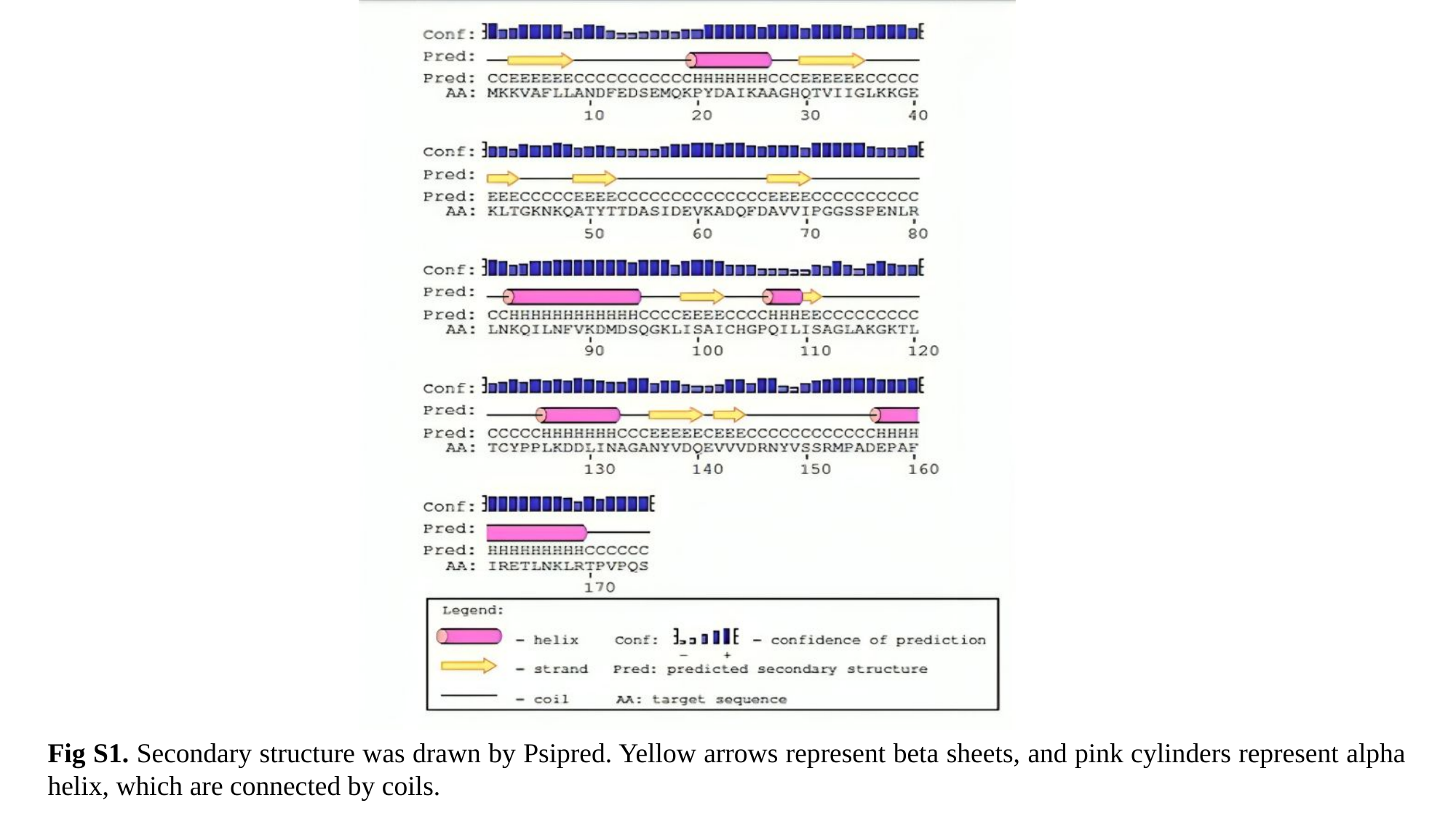

Fig S1. Secondary structure was drawn by Psipred. Yellow arrows represent beta sheets, and pink cylinders represent alpha helix, which are connected by coils.
