# Supplementary material for: Heterologous expression, purification, and biochemical characterization of protease 3075 from Cohnella sp. A01
Source: PLoS One. 2024 Dec 16;19(12):e0310910. doi: 10.1371/journal.pone.0310910 (PMC11649109; doi:10.1371/journal.pone.0310910)
Supplement: S3 Fig — Registering the number one and zero against DB_state indicates the presence and absence of disulfide bonds, respectively. DB_conf represents the confidence factor. The closer the DB_conf number to 9, the higher the confidence level. (PPTX) [file pone.0310910.s003.pptx]

## Slide 1
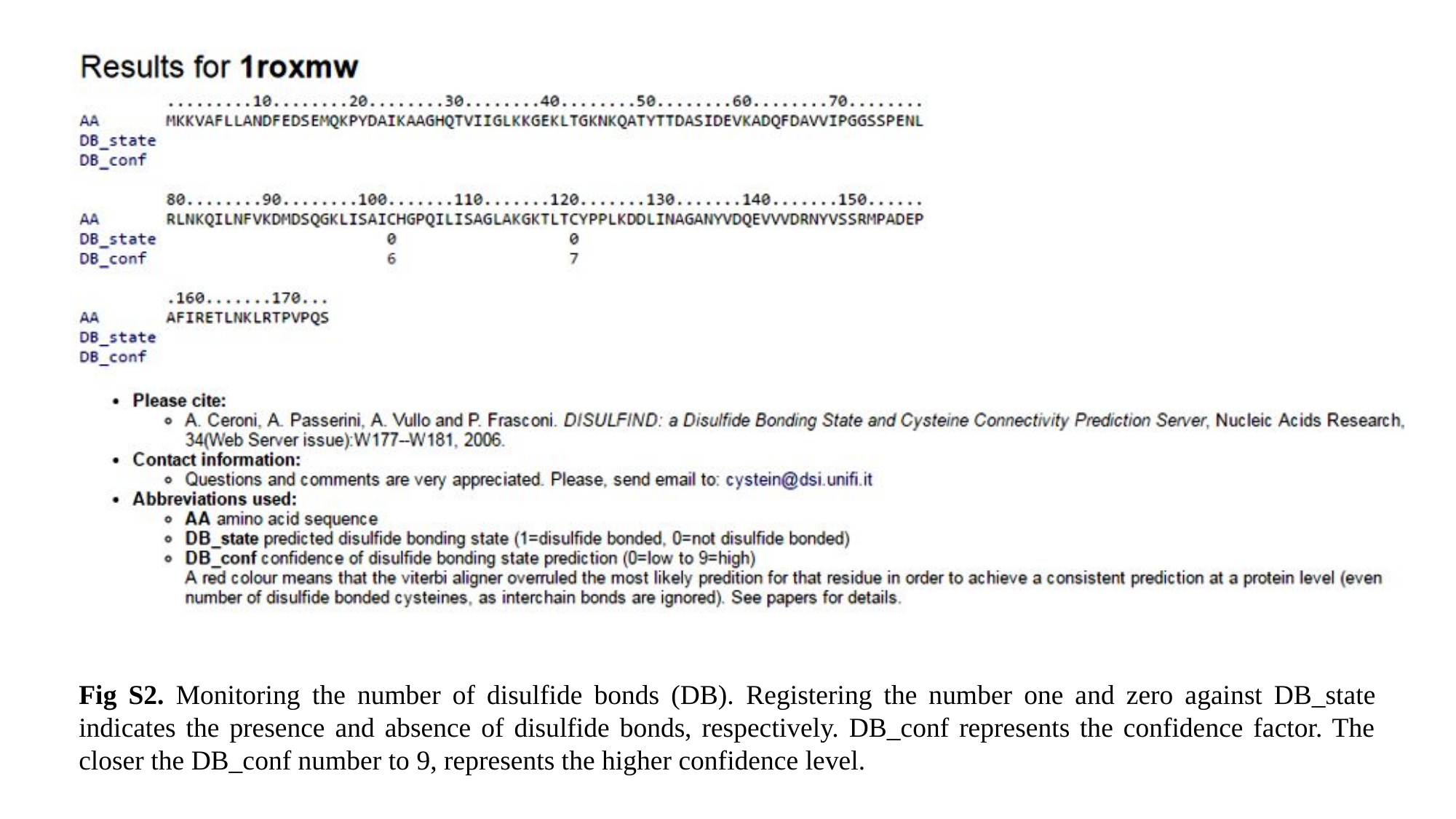

Fig S2. Monitoring the number of disulfide bonds (DB). Registering the number one and zero against DB_state indicates the presence and absence of disulfide bonds, respectively. DB_conf represents the confidence factor. The closer the DB_conf number to 9, represents the higher confidence level.
