# Supplementary figures and images for: Heterologous expression, purification, and biochemical characterization of protease 3075 from Cohnella sp. A01
Source: PLoS One. 2024 Dec 16;19(12):e0310910. doi: 10.1371/journal.pone.0310910 (PMC11649109; doi:10.1371/journal.pone.0310910)

## Slide 1
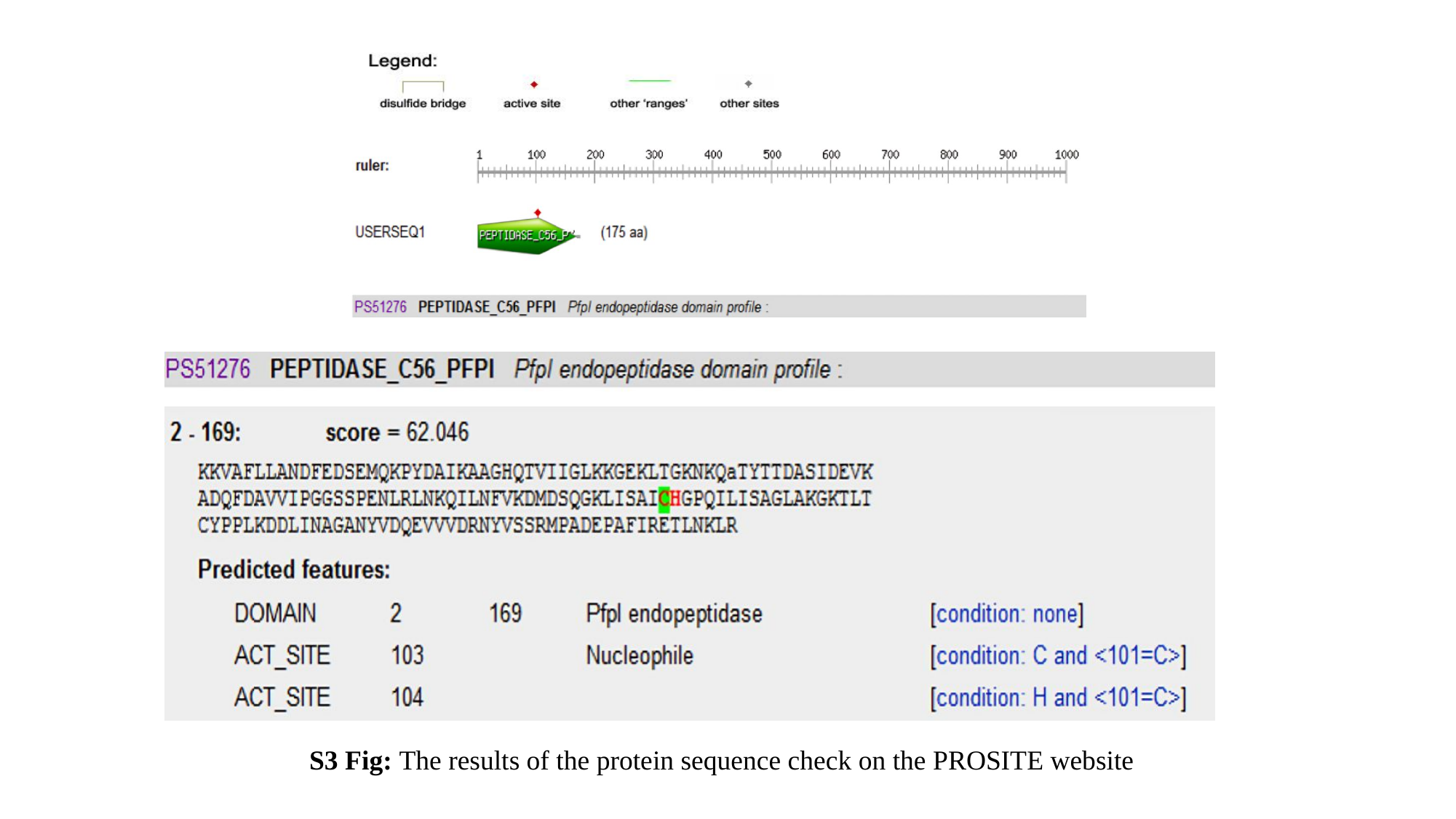

S3 Fig: The results of the protein sequence check on the PROSITE website

Supplement: S4 Fig — (PPTX) [file pone.0310910.s004.pptx]
