# Supplementary material for: Heterologous expression, purification, and biochemical characterization of protease 3075 from Cohnella sp. A01
Source: PLoS One. 2024 Dec 16;19(12):e0310910. doi: 10.1371/journal.pone.0310910 (PMC11649109; doi:10.1371/journal.pone.0310910)
Supplement: S1 Table — (DOCX) [file pone.0310910.s005.docx]

**Table S5:** Comparison of protease 3075 with other industrial proteases in terms of effects on different substrates

| Ref | Gelatin | Azo-albumin | Azo-casein | Casein | Enzyme | Microorganism |
| --- | --- | --- | --- | --- | --- | --- |
| Current study | 80% | 10% | 20% | 100% | Protease | *Cohnella sp.A01* |
| Aruimonim,2007 | 22% | 58% | 85% | 100% | Alkaline protease | *B.stearothermophilus* |
| Shivakumars,2012 | 90% | - | _ | 80% | Acid protease | *Aspergillus Sp.* |
| LbraheemAs,2017 | 112% | - | _ | 100% | Protease | *Citrus sinensi* |
| Yildirimv,2017 | 25% | - | 15% | 100% | Serine protease | *BAeribacillus*  *Pallidus* |
