# Supplementary material for: Heterologous expression, purification, and biochemical characterization of protease 3075 from Cohnella sp. A01
Source: PLoS One. 2024 Dec 16;19(12):e0310910. doi: 10.1371/journal.pone.0310910 (PMC11649109; doi:10.1371/journal.pone.0310910)
Supplement: S2 Table — (DOCX) [file pone.0310910.s006.docx]

**Table S6:** Comparison of some biochemical features of protease 3075 with other proteases

| Ref | Property | Optimum pH | Optimum temperature  (°C) | Concentration (mM) | Molecular weight  kDa)) | Enzyme | Microorganism |
| --- | --- | --- | --- | --- | --- | --- | --- |
| Current study | Thermal stability, increased activity in the presence of Tween80 and acetone | 6 | 70 | 19 | Ca^2+^ | Cysteine protease | *Cohnella sp.A01* |
| JoshiB,2010 | Thermal stability | 9 | 60 | 34 | Ca^2+^ | Alkaline protease | *Bacillus ﬁrmus Tap5* |
| HsiaoN,2014 | activity in the presence of Ca2+ | 4 | 70 | 39 | Ca^2+^ | Aspartic protease | *Rhizopus oryzae* |
| Thremacoldic,2007 | Ca2+ increases thermal stability | 5.9 | 40 | 35 | Ca^2+^ | Alkaline protease | *Aspergillus clavatus* |
| AmidM,2014 | Increased activity in the presence of Ca2+ ion and stability in the presence of Tween80 and acetone | 8 | 70 | 27 | Ca^2+^ | Alkaline protease | *Hylocereus polyrhizus* |
