# Supplementary material for: Heterologous expression, purification, and biochemical characterization of protease 3075 from Cohnella sp. A01
Source: PLoS One. 2024 Dec 16;19(12):e0310910. doi: 10.1371/journal.pone.0310910 (PMC11649109; doi:10.1371/journal.pone.0310910)
Supplement: S3 Table — (DOCX) [file pone.0310910.s007.docx]

**Table S7**: sequence similarity analysis of protease 3075

| **Select for downloading or viewing reports** | Description | Scientific Name | Max Score | | Total Score | Query Cover | E value | Per. Ident | | Acc. Len | | Accession |
| --- | --- | --- | --- | --- | --- | --- | --- | --- | --- | --- | --- | --- |
| **Select seq ref\|WP_027092884.1\|** | type 1 glutamine amidotransferase domain-containing protein [Cohnella thermotolerans] | Cohnella thermotolerans | | 355 | 355 | 100% | 0/00000000000000000 | | 98.86% | 175 | WP_027092884.1 | |
| **Select seq ref\|WP_028988075.1\|** | type 1 glutamine amidotransferase domain-containing protein [Thermicanus aegyptius] | Thermicanus aegyptius | | 347 | 347 | 100% | 0/00000000000000000 | | 96.57% | 175 | WP_028988075.1 | |
| **Select seq ref\|WP_067935194.1\|** | type 1 glutamine amidotransferase domain-containing protein [Alicyclobacillus kakegawensis] | Alicyclobacillus kakegawensis | | 332 | 332 | 100% | 0/00000000000000000 | | 91.43% | 175 | WP_067935194.1 | |
| **Select seq ref\|WP_273367104.1\|** | type 1 glutamine amidotransferase domain-containing protein [Alicyclobacillus herbarius] | Alicyclobacillus herbarius | | 331 | 331 | 99% | 0/00000000000000000 | | 91.38% | 175 | WP_273367104.1 | |
| **Select seq ref\|WP_268004548.1\|** | type 1 glutamine amidotransferase domain-containing protein [Alicyclobacillus fastidiosus] | Alicyclobacillus fastidiosus | | 330 | 330 | 100% | 0/00000000000000000 | | 91.43% | 175 | WP_268004548.1 | |
| **Select seq ref\|WP_026960963.1\|** | type 1 glutamine amidotransferase domain-containing protein [Alicyclobacillus herbarius] | Alicyclobacillus herbarius | | 330 | 330 | 99% | 0/00000000000000000 | | 90.80% | 175 | WP_026960963.1 | |
| **Select seq ref\|WP_166157022.1\|** | type 1 glutamine amidotransferase domain-containing protein [Paenibacillus agricola] | Paenibacillus agricola | | 329 | 329 | 100% | 0/00000000000000000 | | 92.00% | 177 | WP_166157022.1 | |
| **Select seq ref\|WP_029420655.1\|** | type 1 glutamine amidotransferase domain-containing protein [Alicyclobacillus macrosporangiidus] | Alicyclobacillus macrosporangiidus | | 328 | 328 | 100% | 0/00000000000000000 | | 90.29% | 175 | WP_029420655.1 | |
| **Select seq ref\|WP_054795277.1\|** | type 1 glutamine amidotransferase domain-containing protein [Paenibacillus] | Paenibacillus | | 327 | 327 | 99% | 0/00000000000000000 | | 91.95% | 175 | WP_054795277.1 | |
| **Select seq ref\|WP_268046243.1\|** | type 1 glutamine amidotransferase domain-containing protein [Alicyclobacillus dauci] | Alicyclobacillus dauci | | 324 | 324 | 100% | 0/00000000000000000 | | 89.14% | 175 | WP_268046243.1 | |
| **Select seq ref\|WP_019004749.1\|** | type 1 glutamine amidotransferase domain-containing protein [Cohnella laeviribosi] | Cohnella laeviribosi | | 320 | 320 | 96% | 0/00000000000000000 | | 91.67% | 169 | WP_019004749.1 | |
| **Select seq ref\|WP_100667354.1\|** | type 1 glutamine amidotransferase domain-containing protein [Kyrpidia spormannii] | Kyrpidia spormannii | | 309 | 309 | 99% | 0/00000000000000000 | | 81.61% | 176 | WP_100667354.1 | |
| **Select seq ref\|WP_288010633.1\|** | type 1 glutamine amidotransferase domain-containing protein [Kyrpidia sp.] | Kyrpidia sp. | | 309 | 309 | 99% | 0/00000000000000000 | | 81.61% | 176 | WP_288010633.1 | |
| **Select seq ref\|WP_159881799.1\|** | type 1 glutamine amidotransferase domain-containing protein [Paenibacillus puerhi] | Paenibacillus puerhi | | 284 | 284 | 99% | 0/00000000000000000 | | 77.59% | 175 | WP_159881799.1 | |
| **Select seq ref\|WP_072328401.1\|** | type 1 glutamine amidotransferase domain-containing protein [unclassified Paenibacillus] | unclassified Paenibacillus | | 278 | 278 | 99% | 0/00000000000000000 | | 78.16% | 175 | WP_072328401.1 | |
| **Select seq ref\|WP_341415250.1\|** | type 1 glutamine amidotransferase domain-containing protein [Paenibacillus filicis] | Paenibacillus filicis | | 276 | 276 | 99% | 0/00000000000000000 | | 75.86% | 175 | WP_341415250.1 | |
| **Select seq ref\|WP_076357694.1\|** | type 1 glutamine amidotransferase domain-containing protein [unclassified Paenibacillus] | unclassified Paenibacillus | | 256 | 256 | 100% | 0/00000000000000000 | | 68.57% | 177 | WP_076357694.1 | |
| **Select seq emb\|CDN44479.1\|** | Protein YhbO [Paenibacillus sp. P22] | Paenibacillus sp. P22 | | 255 | 255 | 100% | 0/00000000000000000 | | 68.57% | 179 | CDN44479.1 | |
| **Select seq ref\|WP_048748138.1\|** | type 1 glutamine amidotransferase domain-containing protein [Paenibacillus] | Paenibacillus | | 254 | 254 | 100% | 0/00000000000000000 | | 68.57% | 177 | WP_048748138.1 | |
| **Select seq ref\|WP_058304320.1\|** | type 1 glutamine amidotransferase domain-containing protein [Gorillibacterium timonense] | Gorillibacterium timonense | | 253 | 253 | 99% | 0/00000000000000000 | | 66.67% | 174 | WP_058304320.1 | |
| **Select seq emb\|SDS94415.1\|** | protease I [Paenibacillaceae bacterium GAS479] | Paenibacillaceae bacterium GAS479 | | 253 | 253 | 100% | 0/00000000000000000 | | 67.43% | 177 | SDS94415.1 | |
| **Select seq ref\|WP_089525052.1\|** | type 1 glutamine amidotransferase domain-containing protein [Paenibacillus herberti] | Paenibacillus herberti | | 252 | 252 | 100% | 0/00000000000000000 | | 67.43% | 177 | WP_089525052.1 | |
| **Select seq ref\|WP_028598913.1\|** | type 1 glutamine amidotransferase domain-containing protein [Paenibacillus] | Paenibacillus | | 251 | 251 | 100% | 0/00000000000000000 | | 68.57% | 177 | WP_028598913.1 | |
| **Select seq gb\|UUZ83416.1\|** | type 1 glutamine amidotransferase [Paenibacillus sp. P26] | Paenibacillus sp. P26 | | 251 | 251 | 97% | 0/00000000000000000 | | 68.24% | 176 | UUZ83416.1 | |
| **Select seq ref\|WP_315607641.1\|** | type 1 glutamine amidotransferase domain-containing protein [Paenibacillus aurantius] | Paenibacillus aurantius | | 249 | 249 | 96% | 0/00000000000000000 | | 69.64% | 170 | WP_315607641.1 | |
| **Select seq ref\|WP_168909413.1\|** | type 1 glutamine amidotransferase domain-containing protein [Paenibacillus albicereus] | Paenibacillus albicereus | | 250 | 250 | 100% | 0/00000000000000000 | | 68.00% | 177 | WP_168909413.1 | |
| **Select seq ref\|WP_331849018.1\|** | type 1 glutamine amidotransferase domain-containing protein [Paenibacillus sp. M1] | Paenibacillus sp. M1 | | 249 | 249 | 96% | 0/00000000000000000 | | 71.43% | 168 | WP_331849018.1 | |
| **Select seq ref\|WP_165095246.1\|** | type 1 glutamine amidotransferase domain-containing protein [Paenibacillus apii] | Paenibacillus apii | | 248 | 248 | 99% | 0/00000000000000000 | | 67.82% | 174 | WP_165095246.1 | |
| **Select seq ref\|WP_339278993.1\|** | type 1 glutamine amidotransferase domain-containing protein [Paenibacillus sp. FSL W8-1187] | Paenibacillus sp. FSL W8-1187 | | 248 | 248 | 100% | 0/00000000000000000 | | 68.00% | 177 | WP_339278993.1 | |
| **Select seq ref\|WP_127200477.1\|** | type 1 glutamine amidotransferase domain-containing protein [Paenibacillus zeisoli] | Paenibacillus zeisoli | | 248 | 248 | 96% | 0/00000000000000000 | | 70.83% | 169 | WP_127200477.1 | |
| **Select seq ref\|WP_025336000.1\|** | type 1 glutamine amidotransferase domain-containing protein [Paenibacillus sabinae] | Paenibacillus sabinae | | 248 | 248 | 99% | 0/00000000000000000 | | 67.82% | 174 | WP_025336000.1 | |
| **Select seq ref\|WP_213618212.1\|** | type 1 glutamine amidotransferase domain-containing protein [Paenibacillus sp. J22TS3] | Paenibacillus sp. J22TS3 | | 246 | 246 | 96% | 0/00000000000000000 | | 69.05% | 169 | WP_213618212.1 | |
| **Select seq ref\|WP_251561124.1\|** | type 1 glutamine amidotransferase domain-containing protein [Paenibacillus pasadenensis] | Paenibacillus pasadenensis | | 246 | 246 | 100% | 0/00000000000000000 | | 66.29% | 177 | WP_251561124.1 | |
| **Select seq ref\|WP_089198130.1\|** | type 1 glutamine amidotransferase domain-containing protein [Paenibacillus xerothermodurans] | Paenibacillus xerothermodurans | | 246 | 246 | 97% | 0/00000000000000000 | | 67.06% | 176 | WP_089198130.1 | |
| **Select seq ref\|WP_223083999.1\|** | type 1 glutamine amidotransferase domain-containing protein [Paenibacillus] | Paenibacillus | | 246 | 246 | 99% | 0/00000000000000000 | | 66.67% | 174 | WP_223083999.1 | |
| **Select seq ref\|WP_124695789.1\|** | type 1 glutamine amidotransferase domain-containing protein [Paenibacillus rhizophilus] | Paenibacillus rhizophilus | | 246 | 246 | 99% | 0/00000000000000000 | | 67.24% | 174 | WP_124695789.1 | |
| **Select seq ref\|WP_045670048.1\|** | type 1 glutamine amidotransferase domain-containing protein [Paenibacillus beijingensis] | Paenibacillus beijingensis | | 245 | 245 | 99% | 0/00000000000000000 | | 67.24% | 176 | WP_045670048.1 | |
| **Select seq ref\|WP_210020799.1\|** | type 1 glutamine amidotransferase domain-containing protein [unclassified Paenibacillus] | unclassified Paenibacillus | | 244 | 244 | 97% | 0/00000000000000000 | | 67.65% | 176 | WP_210020799.1 | |
| **Select seq ref\|WP_068615863.1\|** | type 1 glutamine amidotransferase domain-containing protein [Paenibacillus tuaregi] | Paenibacillus tuaregi | | 244 | 244 | 96% | 0/00000000000000000 | | 68.45% | 169 | WP_068615863.1 | |
| **Select seq ref\|WP_211020128.1\|** | type 1 glutamine amidotransferase domain-containing protein [Paenibacillus] | Paenibacillus | | 244 | 244 | 96% | 0/00000000000000000 | | 68.45% | 168 | WP_211020128.1 | |
| **Select seq ref\|WP_038697921.1\|** | type 1 glutamine amidotransferase domain-containing protein [Paenibacillus stellifer] | Paenibacillus stellifer | | 243 | 243 | 98% | 0/00000000000000000 | | 65.70% | 176 | WP_038697921.1 | |
| **Select seq ref\|WP_138495690.1\|** | type 1 glutamine amidotransferase domain-containing protein [Paenibacillus pinistramenti] | Paenibacillus pinistramenti | | 242 | 242 | 96% | 0/00000000000000000 | | 67.86% | 168 | WP_138495690.1 | |
| **Select seq ref\|WP_036594260.1\|** | type 1 glutamine amidotransferase domain-containing protein [Paenibacillus sophorae] | Paenibacillus sophorae | | 242 | 242 | 98% | 0/00000000000000000 | | 65.70% | 174 | WP_036594260.1 | |
| **Select seq ref\|WP_179036676.1\|** | type 1 glutamine amidotransferase domain-containing protein [Paenibacillus sp. URB8-2] | Paenibacillus sp. URB8-2 | | 242 | 242 | 99% | 0/00000000000000000 | | 66.67% | 174 | WP_179036676.1 | |
| **Select seq ref\|WP_341114595.1\|** | type 1 glutamine amidotransferase domain-containing protein [Paenibacillus sp. FSL F4-0125] | Paenibacillus sp. FSL F4-0125 | | 242 | 242 | 96% | 0/00000000000000000 | | 66.86% | 169 | WP_341114595.1 | |
| **Select seq ref\|WP_251377918.1\|** | type 1 glutamine amidotransferase domain-containing protein [Paenibacillus sp. YPG26] | Paenibacillus sp. YPG26 | | 242 | 242 | 96% | 0/00000000000000000 | | 68.45% | 169 | WP_251377918.1 | |
| **Select seq ref\|WP_018887753.1\|** | type 1 glutamine amidotransferase domain-containing protein [Paenibacillus massiliensis] | Paenibacillus massiliensis | | 241 | 241 | 96% | 0/00000000000000000 | | 66.07% | 168 | WP_018887753.1 | |
| **Select seq ref\|WP_341179761.1\|** | type 1 glutamine amidotransferase domain-containing protein [Paenibacillus sp. FSL K6-1230] | Paenibacillus sp. FSL K6-1230 | | 241 | 241 | 96% | 0/00000000000000000 | | 66.07% | 168 | WP_341179761.1 | |
| **Select seq ref\|WP_068697146.1\|** | type 1 glutamine amidotransferase domain-containing protein [Paenibacillus yonginensis] | Paenibacillus yonginensis | | 241 | 241 | 96% | 0/00000000000000000 | | 67.26% | 168 | WP_068697146.1 | |
| **Select seq ref\|WP_114494448.1\|** | type 1 glutamine amidotransferase domain-containing protein [Fontibacillus phaseoli] | Fontibacillus phaseoli | | 241 | 241 | 97% | 0/00000000000000000 | | 66.47% | 173 | WP_114494448.1 | |
| **Select seq ref\|WP_173128652.1\|** | type 1 glutamine amidotransferase domain-containing protein [Paenibacillus tritici] | Paenibacillus tritici | | 241 | 241 | 96% | 0/00000000000000000 | | 69.05% | 168 | WP_173128652.1 | |
| **Select seq ref\|WP_094093892.1\|** | type 1 glutamine amidotransferase domain-containing protein [Paenibacillus physcomitrellae] | Paenibacillus physcomitrellae | | 240 | 240 | 96% | 0/00000000000000000 | | 66.67% | 168 | WP_094093892.1 | |
| **Select seq ref\|WP_213508793.1\|** | type 1 glutamine amidotransferase domain-containing protein [Paenibacillus faecis] | Paenibacillus faecis | | 240 | 240 | 96% | 0/00000000000000000 | | 67.26% | 168 | WP_213508793.1 | |
| **Select seq ref\|WP_341045949.1\|** | type 1 glutamine amidotransferase domain-containing protein [Paenibacillus sp. FSL R5-0407] | Paenibacillus sp. FSL R5-0407 | | 240 | 240 | 96% | 0/00000000000000000 | | 67.86% | 168 | WP_341045949.1 | |
| **Select seq ref\|WP_042130474.1\|** | type 1 glutamine amidotransferase domain-containing protein [Paenibacillus] | Paenibacillus | | 240 | 240 | 96% | 0/00000000000000000 | | 66.27% | 169 | WP_042130474.1 | |
| **Select seq ref\|WP_042191095.1\|** | type 1 glutamine amidotransferase domain-containing protein [Paenibacillus sp. FSL H7-0737] | Paenibacillus sp. FSL H7-0737 | | 240 | 240 | 96% | 0/00000000000000000 | | 66.27% | 169 | WP_042191095.1 | |
| **Select seq ref\|WP_341124330.1\|** | type 1 glutamine amidotransferase domain-containing protein [Paenibacillus sp. FSL F4-0236] | Paenibacillus sp. FSL F4-0236 | | 240 | 240 | 96% | 0/00000000000000000 | | 66.27% | 169 | WP_341124330.1 | |
| **Select seq ref\|WP_336102555.1\|** | type 1 glutamine amidotransferase domain-containing protein [Paenibacillus] | Paenibacillus | | 239 | 239 | 96% | 0/00000000000000000 | | 67.86% | 168 | WP_336102555.1 | |
| **Select seq ref\|WP_340752160.1\|** | type 1 glutamine amidotransferase domain-containing protein [Paenibacillus sp. FSL R7-0204] | Paenibacillus sp. FSL R7-0204 | | 239 | 239 | 96% | 0/00000000000000000 | | 67.86% | 168 | WP_340752160.1 | |
| **Select seq ref\|WP_339798118.1\|** | type 1 glutamine amidotransferase domain-containing protein [Paenibacillus sp. FSL R5-0744] | Paenibacillus sp. FSL R5-0744 | | 239 | 239 | 96% | 0/00000000000000000 | | 66.27% | 169 | WP_339798118.1 | |
| **Select seq ref\|WP_110931802.1\|** | type 1 glutamine amidotransferase domain-containing protein [Paenibacillus bouchesdurhonensis] | Paenibacillus bouchesdurhonensis | | 239 | 239 | 96% | 0/00000000000000000 | | 67.86% | 168 | WP_110931802.1 | |
| **Select seq ref\|WP_339311230.1\|** | type 1 glutamine amidotransferase domain-containing protein [Paenibacillus sp. FSL M7-0896] | Paenibacillus sp. FSL M7-0896 | | 238 | 238 | 96% | 0/00000000000000000 | | 68.45% | 168 | WP_339311230.1 | |
| **Select seq ref\|WP_094873671.1\|** | type 1 glutamine amidotransferase domain-containing protein [unclassified Paenibacillus] | unclassified Paenibacillus | | 238 | 238 | 96% | 0/00000000000000000 | | 65.68% | 169 | WP_094873671.1 | |
| **Select seq ref\|WP_108466612.1\|** | type 1 glutamine amidotransferase domain-containing protein [Paenibacillus sp. CAA11] | Paenibacillus sp. CAA11 | | 238 | 238 | 96% | 0/00000000000000000 | | 65.68% | 169 | WP_108466612.1 | |
| **Select seq ref\|WP_149647657.1\|** | type 1 glutamine amidotransferase domain-containing protein [unclassified Paenibacillus] | unclassified Paenibacillus | | 238 | 238 | 96% | 0/00000000000000000 | | 65.68% | 169 | WP_149647657.1 | |
| **Select seq ref\|WP_042139537.1\|** | type 1 glutamine amidotransferase domain-containing protein [Paenibacillus sp. FSL P4-0081] | Paenibacillus sp. FSL P4-0081 | | 238 | 238 | 96% | 0/00000000000000000 | | 67.26% | 168 | WP_042139537.1 | |
| **Select seq ref\|WP_171720879.1\|** | type 1 glutamine amidotransferase domain-containing protein [Paenibacillus phytohabitans] | Paenibacillus phytohabitans | | 238 | 238 | 96% | 0/00000000000000000 | | 67.86% | 168 | WP_171720879.1 | |
| **Select seq ref\|WP_042238705.1\|** | type 1 glutamine amidotransferase domain-containing protein [unclassified Paenibacillus] | unclassified Paenibacillus | | 238 | 238 | 96% | 0/00000000000000000 | | 67.86% | 168 | WP_042238705.1 | |
| **Select seq ref\|WP_076193216.1\|** | type 1 glutamine amidotransferase domain-containing protein [Paenibacillus odorifer] | Paenibacillus odorifer | | 238 | 238 | 96% | 0/00000000000000000 | | 65.68% | 169 | WP_076193216.1 | |
| **Select seq ref\|WP_076284837.1\|** | type 1 glutamine amidotransferase domain-containing protein [Paenibacillus] | Paenibacillus | | 238 | 238 | 96% | 0/00000000000000000 | | 65.68% | 169 | WP_076284837.1 | |
| **Select seq ref\|WP_238653425.1\|** | type 1 glutamine amidotransferase domain-containing protein [Paenibacillus piscarius] | Paenibacillus piscarius | | 238 | 238 | 96% | 0/00000000000000000 | | 69.05% | 168 | WP_238653425.1 | |
| **Select seq ref\|WP_334075650.1\|** | type 1 glutamine amidotransferase domain-containing protein [unclassified Paenibacillus] | unclassified Paenibacillus | | 238 | 238 | 96% | 0/00000000000000000 | | 66.67% | 168 | WP_334075650.1 | |
| **Select seq ref\|WP_076141540.1\|** | type 1 glutamine amidotransferase domain-containing protein [Paenibacillus] | Paenibacillus | | 238 | 238 | 96% | 0/00000000000000000 | | 66.27% | 169 | WP_076141540.1 | |
| **Select seq ref\|WP_076245164.1\|** | type 1 glutamine amidotransferase domain-containing protein [Paenibacillus sp. FSL H8-0259] | Paenibacillus sp. FSL H8-0259 | | 238 | 238 | 96% | 0/00000000000000000 | | 67.86% | 168 | WP_076245164.1 | |
| **Select seq ref\|WP_036690533.1\|** | type 1 glutamine amidotransferase domain-containing protein [unclassified Paenibacillus] | unclassified Paenibacillus | | 237 | 237 | 96% | 0/00000000000000000 | | 66.67% | 168 | WP_036690533.1 | |
| **Select seq ref\|WP_339157573.1\|** | type 1 glutamine amidotransferase domain-containing protein [Paenibacillus sp. FSL W8-0186] | Paenibacillus sp. FSL W8-0186 | | 237 | 237 | 96% | 0/00000000000000000 | | 66.86% | 169 | WP_339157573.1 | |
| **Select seq ref\|WP_076297543.1\|** | type 1 glutamine amidotransferase domain-containing protein [Paenibacillus] | Paenibacillus | | 237 | 237 | 96% | 0/00000000000000000 | | 66.27% | 169 | WP_076297543.1 | |
| **Select seq ref\|WP_339243269.1\|** | type 1 glutamine amidotransferase domain-containing protein [Paenibacillus sp. FSL F4-0243] | Paenibacillus sp. FSL F4-0243 | | 237 | 237 | 96% | 0/00000000000000000 | | 65.09% | 169 | WP_339243269.1 | |
| **Select seq ref\|WP_209872297.1\|** | type 1 glutamine amidotransferase domain-containing protein [Paenibacillus] | Paenibacillus | | 237 | 237 | 96% | 0/00000000000000000 | | 67.86% | 168 | WP_209872297.1 | |
| **Select seq ref\|WP_341129017.1\|** | type 1 glutamine amidotransferase domain-containing protein [Paenibacillus sp. FSL H7-0714] | Paenibacillus sp. FSL H7-0714 | | 237 | 237 | 96% | 0/00000000000000000 | | 65.68% | 169 | WP_341129017.1 | |
| **Select seq gb\|MED5020506.1\|** | type 1 glutamine amidotransferase [Paenibacillus chibensis] | Paenibacillus chibensis | | 237 | 237 | 96% | 0/00000000000000000 | | 67.26% | 168 | MED5020506.1 | |
| **Select seq ref\|WP_039294511.1\|** | type 1 glutamine amidotransferase domain-containing protein [Paenibacillus sp. IHB B 3415] | Paenibacillus sp. IHB B 3415 | | 237 | 237 | 96% | 0/00000000000000000 | | 66.67% | 168 | WP_039294511.1 | |
| **Select seq ref\|WP_213589570.1\|** | type 1 glutamine amidotransferase domain-containing protein [Paenibacillus woosongensis] | Paenibacillus woosongensis | | 237 | 237 | 96% | 0/00000000000000000 | | 66.86% | 169 | WP_213589570.1 | |
| **Select seq ref\|WP_160499036.1\|** | type 1 glutamine amidotransferase domain-containing protein [Paenibacillus dendrobii] | Paenibacillus dendrobii | | 237 | 237 | 96% | 0/00000000000000000 | | 66.67% | 168 | WP_160499036.1 | |
| **Select seq ref\|WP_339266674.1\|** | type 1 glutamine amidotransferase domain-containing protein [Paenibacillus sp. FSL R5-0470] | Paenibacillus sp. FSL R5-0470 | | 237 | 237 | 96% | 0/00000000000000000 | | 65.68% | 169 | WP_339266674.1 | |
| **Select seq ref\|WP_283908727.1\|** | type 1 glutamine amidotransferase domain-containing protein [Paenibacillus sp. G2S3] | Paenibacillus sp. G2S3 | | 237 | 237 | 96% | 0/00000000000000000 | | 65.09% | 169 | WP_283908727.1 | |
| **Select seq tpg\|HEY4432243.1\|** | TPA: type 1 glutamine amidotransferase domain-containing protein [Paenibacillus sp.] | Paenibacillus sp. | | 237 | 237 | 96% | 0/00000000000000000 | | 66.27% | 169 | HEY4432243.1 | |
| **Select seq ref\|WP_018975851.1\|** | type 1 glutamine amidotransferase domain-containing protein [Saccharibacillus kuerlensis] | Saccharibacillus kuerlensis | | 237 | 237 | 96% | 0/00000000000000000 | | 65.48% | 168 | WP_018975851.1 | |
| **Select seq ref\|WP_340985676.1\|** | type 1 glutamine amidotransferase domain-containing protein [unclassified Paenibacillus] | unclassified Paenibacillus | | 237 | 237 | 96% | 0/00000000000000000 | | 67.86% | 168 | WP_340985676.1 | |
| **Select seq ref\|WP_340950716.1\|** | type 1 glutamine amidotransferase domain-containing protein [Paenibacillus sp. FSL R7-0210] | Paenibacillus sp. FSL R7-0210 | | 236 | 236 | 96% | 0/00000000000000000 | | 67.26% | 168 | WP_340950716.1 | |
| **Select seq ref\|WP_076281193.1\|** | type 1 glutamine amidotransferase domain-containing protein [Paenibacillus] | Paenibacillus | | 236 | 236 | 96% | 0/00000000000000000 | | 65.68% | 169 | WP_076281193.1 | |
| **Select seq ref\|WP_339250400.1\|** | type 1 glutamine amidotransferase domain-containing protein [Paenibacillus sp. FSL P2-0136] | Paenibacillus sp. FSL P2-0136 | | 236 | 236 | 96% | 0/00000000000000000 | | 67.86% | 168 | WP_339250400.1 | |
| **Select seq ref\|WP_234533436.1\|** | type 1 glutamine amidotransferase domain-containing protein [Paenibacillus pseudetheri] | Paenibacillus pseudetheri | | 236 | 236 | 96% | 0/00000000000000000 | | 64.50% | 169 | WP_234533436.1 | |
| **Select seq ref\|WP_094877950.1\|** | type 1 glutamine amidotransferase domain-containing protein [unclassified Paenibacillus] | unclassified Paenibacillus | | 236 | 236 | 96% | 0/00000000000000000 | | 65.09% | 169 | WP_094877950.1 | |
| **Select seq ref\|WP_155610763.1\|** | type 1 glutamine amidotransferase domain-containing protein [Paenibacillus woosongensis] | Paenibacillus woosongensis | | 236 | 236 | 96% | 0/00000000000000000 | | 66.86% | 169 | WP_155610763.1 | |
| **Select seq ref\|WP_289390232.1\|** | type 1 glutamine amidotransferase domain-containing protein [unclassified Paenibacillus] | unclassified Paenibacillus | | 236 | 236 | 94% | 0/00000000000000000 | | 66.87% | 168 | WP_289390232.1 | |
| **Select seq ref\|WP_150457010.1\|** | type 1 glutamine amidotransferase domain-containing protein [Paenibacillus spiritus] | Paenibacillus spiritus | | 237 | 237 | 98% | 0/00000000000000000 | | 64.16% | 175 | WP_150457010.1 | |
| **Select seq ref\|WP_036677639.1\|** | type 1 glutamine amidotransferase domain-containing protein [Paenibacillus] | Paenibacillus | | 236 | 236 | 96% | 0/00000000000000000 | | 65.68% | 169 | WP_036677639.1 | |
| **Select seq ref\|WP_038572983.1\|** | type 1 glutamine amidotransferase domain-containing protein [Paenibacillus] | Paenibacillus | | 236 | 236 | 96% | 0/00000000000000000 | | 66.27% | 169 | WP_038572983.1 | |
