# Supplementary material for: Heterologous expression, purification, and biochemical characterization of protease 3075 from Cohnella sp. A01
Source: PLoS One. 2024 Dec 16;19(12):e0310910. doi: 10.1371/journal.pone.0310910 (PMC11649109; doi:10.1371/journal.pone.0310910)
Supplement: Attachment — Submitted filename: Rebattle 3075.docx [file pone.0310910.s008.docx]

Please submit your revised manuscript by Aug 24 2024 11:59PM

Reviewer #1

1. The initial part of the abstract is long and unnecessary. The background can be condensed into one set of sentences

Thank you for pointing this out! As mentioned, the background was condensed into one set of sentences.

2. The biochemical and structural aspects of the protease were investigated for which enzyme, the native or recombinant proteases?

Thank you! The biochemical and structural aspects of the protease were investigated for recombinant proteases 3075.

3. The results of the expression should be briefly mentioned in the abstract.

Thank you for pointing this out! We rewrite the abstract part.

4. Tween 80 and acetone increased the enzyme activity by fourfold. However, the sentence in the abstract is unclear and ambiguous.

Thank you! We found and corrected it.

5. A-The sentence in the abstract-----"presence of Tween 80 heightened the stability of the protease structure,” is ambiguous and appears speculative. It needs further clarity.

B- What is the indication of “heightened stability” in the context of the molecule's structure?

A- Thank you! We improved the abstract part.

B- There was a typographical error. We considered and corrected it.

6. The abstract overall needs reorganization and condensation.

Thank you for pointing this out! As mentioned, the abstract part was reorganized.

7. In the methods, why the section, ‘2.2.5. Cohnella sp. A01 cell culture and DNA extraction’ is included at a later stage? In this context, the authors should reconsider the organization and sequence of the contents. Should it be started with bioinformatics and its analysis?

Thank you for pointing this out! As mentioned, we reconsider the organization and sequence of the contents.

8. What is the nature of the protease gene primer? How was it obtained?

Thank you! The nature of the protease gene primer was a deoxyribonucleic acid sequence. The primers were made by the Tekapozist company in Iran.

9. In the table, the number of atoms for the entire protease molecule? What is its significance in the context of structure and function?

Thank you! Generally, the number of atoms in the protein was determined to assign the size and complexity of the protein. Moreover, to perform molecular docking and MD simulation studies it was better to know the number of atoms to design the simulation box.

10. To conclude, the structural stability of the protease in response to its interaction with twin-80 needs to be described in a condensed and cohesive manner.

Thank you! As advised, we described the structural stability of protease 3075 in the presence of Tween-80 in a condensed and cohesive manner (472-475 lines).

11. If certain studies with the native enzymes are also done, a comparison of the enzymatic/biochemical feature should also be discussed.

Thank you for pointing this out! As mentioned in the manuscript (line 394), we comprised the temperature, optimum pH, substrate specificity, and some features of protease 3075 with other proteases. Data added to the supplementary file (S5 and S6 Tables).

12. Some figures are placed at the end, while the related captions are included in the text. It would be better and more convenient if the captions were placed near/below the figures—for instance, Figure S4.

Thank you for pointing this out! Because S4 Fig was related to supplementary files, it was placed in the supplementary file. But as mentioned, we rearranged the other figures According to the mentioned order.

13. Marker bands are fuzzy. Well-3 and 5 are both labeled as digested recombinant plasmid, but their band patterns are different. This requires confirmation and clarity.

Thank you! At that time, the quality of the used camera was not suitable. According to our knowledge, their band patterns are not different. Unfortunately, the gel is now gone and it is not possible to take pictures again. However, we had another photo of the same gel and replaced it.

14. What are the bands inside the well-7 and 8? Are they supposed to be visualized, or is it a false result?

Thank you! There is only one band in each mentioned well. As described in the figure caption, the bands inside wells 7 and 8 represent the PCR colony and PCR plasmid, respectively.

15. Why is the band in well-8, PCR plasmid at lower bp to colony PCR band? Shouldn’t it be at a higher bp?

Thank you! The bands in Wells 7 and 8 are in the same area (525 bp), which is indicated by the red line on the gel image (Fig. 1). However, because, in Well 7 a little bit of the bacterial colony was visually picked up, compared to well 8 (PCR plasmid), its band is only slightly sharper.

**Fig. 1:** Gel electrophoresis.7:PCR colony, 8: PCR plasmid.

16. The arrangement of figures and their captions are scattered.

Thank you for pointing this out! As mentioned in the answer to question 12, We rearranged the figures.

17. Some specific distinctions regarding structure-function, gene cloning and expression, and nobility between this enzyme and others described so far should be highlighted in the highlights and the conclusion/abstract. Otherwise, the study would appear as routine. Some more recent work on these aspects should also be included, specifically from extremophiles.

Thank you for pointing this out! We improved the abstract and conclusion parts.

18. The manuscript over all needs careful proofreading for typographical errors and improvement in language and expression.

Thank you! We found typographical errors and improved the language of the manuscript.

Reviewer #2:

1. The ms should clearly be edited by an English spoken person.

Thank you! We improved the language of the manuscript.

2. The authors should indicate the source of Cohnella sp.A01 in the manuscript.

Thank you! The bacterial genome of Cohnella sp. A01 was isolated from a shrimp breeding pond in Abadan, Iran. This sequence was determined by Baseclear Netherlands company and its possible genes were predicted

3. Tween 80 is a commonly used surfactant and it can stabilize proteins and improve the catalytic activity of enzymes. What is the significance of the molecular docking between Tween 80 and protease AND MD?

Thank you ! As mentioned in the manuscript (423-425 lines), since Tween 80 had the had best binding energy and the greatest effect on the enzyme among the examined ligands, docking studies, and enzyme molecular simulation were performed in the presence of the mentioned ligand.

4. The unit format in the article should be uniform. e.g. Line 162 “ng/μl” , Line 212 “ mg.ml-1”)

Thank you for pointing this out! All unit formats were uniform.

5. Line 212, 213 “ mg.ml-1” should be “ mg·ml-1”

Thank you! We found and corrected it.

6. K and V in Km, Vmax, Kcat should be italicized

Thank you for pointing this out! We italicized  *K*_m_*, V*_max_*, and K*_cat_.

7. Line 230 “ The reaction mixture was incubated for 3 hours at 4 ºC.” Why did the author choose 4 ºC?

Thank you! In general, after the preparation of the reaction mixture, to prevent the loss of activity or any type of additional reaction, the mixture was kept at 4 ºC.

8. Section 3.1.1. Should provide sequence similarity analysis of protease 3075.

Thank you for pointing this out! Sequence similarity analysis of protease 3075 prepared in Excel format in a supplementary file.

9. Fig or Fig. ? It should be unified

Thank you for pointing this out! We found and unified its writing format.

10. Section 3.4. The specific activity of protease 3075 should be provided. Specific enzyme activity is the number of enzyme units per ml divided by the concentration of protein in mg/ml.

Thank you! Specific activity values for crud extract and after Ni-NTA are reported in Table 1.

| Total Protein (mg.ml^-1^) | Total Activity (U.ml^-1^) | Specific Activity (U.mg^-1^ protein) |
| --- | --- | --- |
| **11.2** | **3.92** | **3.92/11.2= 0.35** |
| **3.6** | **1.44** | **1.44/3.6= 0.4** |

11. Table 7, Why some substances that strongly inhibit enzyme activity at low concentrations are less so at high concentrations?

Thank you for pointing this out! You are right. There was a typographical error. We considered and corrected it.

12. None of the pictures are clear enough.

Thank you! All experimental figures were plotted by sigma plot software. Figures were saved in TIFF format and 600 dpi. Moreover, bioinformatic figures were exported from the software.
